# The correlation between Interleukin 1 β (IL-1β) as an inflammatory marker and Malondialdehyde (MDA) as a lipid peroxidation marker and the development of cardiac and pancreatic complications in humans suspected to scorpion poisoning

**DOI:** 10.1186/s40360-025-01019-6

**Published:** 2025-12-01

**Authors:** Galila Ragab Mebed, Mahmoud Sami Zakaria, Amr Setouhi, Meriam N. N. Rezk

**Affiliations:** 1 Department of Forensic Medicine and Clinical Toxicology, Faculty of Medicine, Minia University, Minia City, Egypt; 2 Department of Cardiology, Faculty of Medicine, Minia University, Minia City, Egypt

**Keywords:** Scorpion, Cardiac complications, Pancreas, Interleukin 1 β (IL-1β) and Malondialdehyde (MDA)

## Abstract

**Background:**

Scorpion envenomation is a common health problem all over the world. Recent experimental studies on animals were held to prove the correlation between interleukin 1 β (IL-1β), lipid peroxidation and nitric oxide (NO) production in the development of cardiac and pancreatic abnormalities after scorpion venom exposure. The aim of this study is to assess the correlation of lipid peroxidation and IL-1β release and the development of cardiac and pancreatic complications associated with scorpion envenomation in humans which will subsequently evaluate their role in prediction of cardiac or pancreatic complications and help the physicians when to determine to use antioxidants and (IL-1β) receptor blockers in treatment in addition to scorpion antivenom.

**Methods:**

In this study, 88 subjects were recruited from El-Minia university hospital. All the patients admitted with scorpion envenomation diagnosis were enrolled over a period of 12 months; from the 1st of Jan 2021 till the 30th of Jan 2022. After exclusion of 7 patients, the remaining subjects were divided into four groups of subjects according to their cardiac function into two groups: group A with normal cardiac function contained: 30 normal subjects, group B contained 51 subjects who had abnormal cardiac functions. The patients were considered to have abnormal cardiac function if they had at least one or more abnormal cardiac investigations (cardiac troponin I (c TN-I), electrocardiogram (ECG), and/or, trans thoracic echocardiography (TTE)).The same patients were divided into another two groups: group C with normal pancreatic function contained: 36 normal subjects, group D contained 45 subjects who had abnormal pancreatic function. Pancreatic function was considered abnormal if one or both of serum amylase & random blood sugar results weren’t normal.

**Results:**

Our results showed the following: Regarding cardiac abnormality, there were significant statistical differences with both levels of IL-1β and MDA. Regarding pancreatic abnormality, there were insignificant statistical differences with both levels of IL-1β and MDA. Cutoff values were calculated to help health care providers to assess when to introduce such treatments in addition to antivenom to help to counteract these complications and decline the mortality rates.

**Conclusion:**

It was concluded that IL-1β and MDA have significant correlations in the development of cardiac and pancreatic functions abnormalities; the treatments that modify their release or counteract their action may be beneficial.

**Trial registration:**

The research was approved by the ethical committee board of the faculty of medicine, Minia University, with approval number 206: December/2021. Patients or their first-degree relatives (according to the conscious level) were informed by all procedures included in the study and were asked to sign informed consent if they agreed to participate in.

**Clinical trial number:**

Not applicable.

## Introduction

Scorpion envenomation is frequent in tropical and subtropical areas, and it is considered a serious public health issue in the Middle East and northern Sahara [1]. According to the world health organization (WHO), around 1.2 to 1.5 million individuals are suspected to scorpion stings annually, with 3000 to 5000 deaths rate [2]. In Egypt, a study revealed that the percent of scorpion cases was 1.6% of all patients presented to a single central poison control center during the same year [3].

The clinical picture of scorpion envenomation includes localized and systemic symptoms. The most common localized symptoms list included mild local pain, localized pruritus, erythema, inflammation, ecchymosis, severe swelling, blisters, and cellulitis, which may progress to significant skin necrosis at the stung site, while systemic symptoms included priapism, sleepiness, exhaustion, irritability, restlessness, pallor, perspiration, hypothermia, cool extremities with trunk hyperthermia, tachycardia, muscle spasms, and convulsion [4].

One of the most harmful effects of scorpion envenomation is cardiac dysfunction. Cardiopulmonary problems such pulmonary edema and/or cardiogenic shock are the key characteristics of severe cases. The main cause of death in people who have been stung by scorpions is still cardiac dysfunction. Myocardial ischemia, release of catecholamines, and the direct impact of scorpion venom on cardiac fibers are the main three explanations to these cardiac complications [5].

Acute pancreatitis has been documented in people who have been stung with different genres of scorpions. Patients complained about abdominal discomfort, nausea, and vomiting. Elevated serum levels of amylase and lipase were recorded [6].

Inflammation and lipid peroxidation are two key processes involved in the pathogenesis of venom-induced tissue damage in scorpion envenomation. Neurotoxins can cause the release of inflammatory mediators through the neuroendocrine-immunological network by interacting with pattern recognition receptors of the innate immune system [7]. Significant changes in the histological structure of the heart, lungs, and liver are also brought about by the scorpion venom, along with an increase in glutathione levels [8].

The correlation between oxidative stress, lipid peroxidation and inflammatory response in scorpion envenomation was previously explained as oxidative stress brought on by excessive reactive oxygen species (ROS) which plays a significant role in the pathogenesis of scorpion envenomation. Nitric oxide (NO) is produced by infiltrated inflammatory cells in various organs of envenomed animals. It has been proposed that the inducible isorform (type II) of NOS, a significant free radical, functions as a second messenger in processes which cause cytokines-mediated circulatory shock [9].

Clinical studies have detected rise in IL-1β and other pro-inflammatory cytokines in collected sera of subjects with severe scorpion envenomation. Moreover, research declared its corroboration in systemic involvement in human pathology [10].

According to studies on rats, recent studies emphasized that usage of NO inhibitors and anti-inflammatory drugs as dexamethasone is fundamental in abolishing cardiac abnormalities with their correlated moralities and hyperglycemia [11, 12].

However, there was previous evidence in animal models and human cell studies regarding IL-1β signaling which is directly involved in cardiac dysfunction and mortality related to scorpion [11]. There was a research gap between the indications and the efficacy of early administration of IL-1β suppressor agents and if they can reduce these risks.

However, in this setting, it is suggested that oxidative stress and mitochondrial dysfunction are potential contributors to organ damage [12]. There is a research gap in evaluation MDA levels in the context of scorpion poisoning in humans and correlating them with cardiac or pancreatic outcomes.

This study is designed to assess the correlation of lipid peroxidation and IL-1β release and the development of cardiac and pancreatic complications associated with scorpion envenomation in humans which will eventually evaluate their role in prediction of cardiac or pancreatic complications and to help in determining when to initiate antioxidants and (IL-1β) receptor blockers treatment in addition to scorpion antivenom to decline death rates due to these complications.

## Subjects and methods

### Subjects

This study was carried out over a period of 12 months; from the 1st of Jan 2021 till the 30th of Jan 2022 on 88 subjects diagnosed with scorpion poisoning. They were recruited from El-Minia university hospital. All the patients admitted into the hospital at that period with scorpion envenomation diagnosis were enrolled.

### Study design and sample size

The study was conducted on 88 subjects; however, 7 patients were excluded as they had underlying illness which can affect the results. The remaining subjects were divided into two groups of subjects according to their cardiac function into two groups: group A with normal cardiac function contained: 30 normal subjects, group B contained 51 subjects who had abnormal cardiac function. The patients were considered to have abnormal cardiac function if he had at least one or more abnormal cardiac investigations c TN-I, ECG, and/or TTE.

The same patients were divided into another two groups: group C with a normal pancreatic function contained: 36 normal subjects, group D contained 45 subjects who had abnormal pancreatic function. Pancreatic function was considered abnormal if one or both of serum amylase & random blood sugar results weren’t normal.

All patients presenting with scorpion envenomation to Minia university hospital during the study period from the 1st of Jan 2021 till the 30th of Jan 2022 were included in the study. Since the study aimed to include all available cases within this time frame, a formal sample size calculation was not performed. This approach allowed the inclusion of the entire population of patients during the specified period, providing a comprehensive overview of the clinical and biochemical outcomes of scorpion envenomation.

### Inclusion and exclusion criteria

Patients included in the study were those diagnosed with scorpion stings, based on a recent history of scorpion sting, clinical presentation at the time of admission, and documented improvement following anti-venom administration. This criterion ensured that only patients with confirmed scorpion envenomation were studied, allowing accurate assessment of its direct effects.

Patients were excluded if they had a history of cardiac disease, were on cardiac medications, had undergone previous cardiac surgery, or had abnormal cardiac investigations. This was done to avoid confounding, as pre-existing cardiac conditions or treatments could independently affect cardiac outcomes, making it difficult to attribute changes specifically to scorpion envenomation.

Similarly, patients with a history of pancreatic disease, diabetes mellitus, or abnormal pancreatic enzyme levels, or those on anti-diabetic treatment were excluded. This exclusion minimized interference from pre-existing metabolic or pancreatic conditions, ensuring that observed changes in pancreatic enzymes or glucose levels could be reliably attributed to the scorpion sting.

### Ethical committee approval

The research was approved by the ethical committee board of the faculty of medicine, Minia University, with approval number 206:12/2021. Patients or their first-degree relatives (according to the conscious level) were informed by all procedures included in the study and were asked to sign informed consent if they agreed to participate in.

### Data collection

#### Clinical data

For every patient clinical examination was done on admission and included: vital signs including blood pressure (BP), heart rate (HR), and respiratory rate (RR). Furthermore, systemic examination was done to detect any systemic abnormalities correlated to cardiovascular dysfunction including hypertension (BP > 140/90 mmHg) or shock (systolic BP < 90 mmHg), weak pulse, heart rate > 100 beats/minute, respiratory rate > 22breaths/min**)**, the presence of dyspnea and chest secretion were evaluated.

To detect pancreatic manifestations, presence of abdominal discomfort, nausea, and vomiting were recorded.

#### Laboratory investigations

All the patients included in this study had the following investigations on admission: measurement of complete blood count (CBC) serum electrolyte (sodium (Na^+^) and potassium (K^+^)), renal function tests (blood urea nitrogen (BUN) and serum creatinine), random blood sugar (RBS), serum amylase, c TN-I, lipid peroxide MDA, IL-1β.

The Enzyme linked immunosorbent assay (ELISA) kit used to measure human IL-1β, was purchased from the Sandwich-elisa principle, albescence, USA. MDA was measured by a kit purchased from Bio-diagnostic, Egypt.

Cardiac TN-I was measured by Minividas, biomerieux-diagnostics, France, kit. C TN-I was considered normal if less than 0.04 ng/ml, borderline if between 0.04 and 0.39 ng/ml and our patients were considered abnormal if equals or more than 0.4 /ml [13].

Serum amylase was performed by using fixed rate method -galg2-cnp, spectrum diagnostics, Egypt. The reference range was 0–90 U/L according to the kit.

All the investigations were done in Minia university hospital laboratory and interpreted with a clinical pathologist.

#### Cardiac assessment

An Electrocardiography was done on admission by using Electrocardiograph, akai, China. ECG indices like rate, PR interval, QRS complex, T wave, QT interval, were recorded. Changes were recorded and reviewed by a cardiologist and considered positive if they were present in at least 2 adjacent leads.

A Trans-thoracic echocardiography was done on admission using two-dimensional (2D) Echocardiography, color doppler ultrasound, Phillips machine medical system, and over, MA, USA. Measurements were adjusted for age, gender, height and weight and expressed by a cardiologist.

#### Sample collection

About 10 ml of venous blood was withdrawn from each subject by using a disposable plastic syringe was divided as follow: 2 ml in ethylene diamine tetra-acetic acid (EDTA) containing tube for CBC. Four ml on plain tube was left to be clotted for 30 min in the incubator then centrifuged at 3000 rpm for 15 min. The expressed serum was used for determination of renal function tests, serum electrolyte, serum amylase, RBS and c TN-I. The remaining 4 ml was used for other serum investigations which were MDA and IL-1β.

### Statistical analysis

The analysis of the data was carried out using the IBM SPSS (Statistical Package for Social Sciences) version 25 statistical package software. Data were expressed as mean ± standard deviation (SD) and minimum and maximum range for parametric quantitative data and by both number and percentage for qualitative data. Analyses were done between every two groups for parametric quantitative data using independent samples T test, while the Chi-square test was used to compare categorical variables. Correlations between variables were done using Pearson’s correlation. Linear regression was done to different variables with IL-1β and MDA. Receiver Operating Characteristic (ROC Curve) analysis was done for variables predicting Cardiac and pancreatic affection. Predictive value less than 0.05 was considered statistically significant.

## Results

### Demographic data for all patients

Ages range was 13–45 years with mean ± SD equals 22.4 ± 8.5 and most of patients were females with 66.7%. The lag time between stings and hospital arrival ranged between 1.5 and 11 h with mean ± SD 5.4 ± 2.8 h. The most common site for stings in our patients was the lower limb (63%).

### Biochemical investigations and CBC for all patients (Table 1)

**Table 1:** Descriptive analysis of the biochemical investigations at the time of admission

Parameter	Range	Mean ± SD
Hb (gm/dl)	(9.5–12.5)	11.1 ± 0.9
TLC (cell/mm^3^)	(4–80)	11.5 ± 13.9
PLT (cell/mm^3^)	(150–500)	306.2 ± 121.6
Na^+^ (mmol/L)	(129–141)	134.3 ± 3.3
K^+^ (mmol/L)	(2.5–4.2)	3.3 ± 0.4
Urea (mg/dl)	(16–50)	30.3 ± 10.3
Creatinine (mg/dl)	(0.5-1)	0.7 ± 0.1
C TN-I (ng/ml)	(1-302)	75.9 ± 98.1
Serum amylase (U/l)	(53–110)	83.7 ± 15.8
RBS (mg/ dl)	(133–400)	230 ± 73.5
IL-1β (pg/ml)	(46.7–89.3)	67.9 ± 14
MDA (nmol/ml)	(1.5–3.8)	2.5 ± 0.6

-Na^+^: Sodium, K^+^: Potassium, HB: Hemoglobin, TLC: Total leucocytic count, PLT: Platelets, C TN-I: Cardiac troponin I, RBS: Random blood sugar, IL-1β: Interleukin-1β, MDA: Malondialdehyde

### Cardiac assessment (Table 2)

**Table 2:** Descriptive analysis of ECG & echocardiography

Parameter	Normal	Abnormal
ECG (n)	30 (37%)	51 (63%)
Echo (n)	54 (66.7%)	27 (33.3%)

-ECG: Electrocardiogram, Echo: Echocardiography

#### Pearson’s correlation between level of IL-1β, MDA and patient demographic data, clinical examination including vital signs, GCS, biochemical investigations and cardiac & pancreatic functions

There were significant statistical differences between level of IL-1β and patients’ DBP, pulse, GCS, Na^+^, K^+^, c TN-I and serum creatinine.

There were significant statistical differences between level of MDA and RR, pulse and GCS, serum amylase, Na^+^, K^+^, c TN-I, serum creatinine and PLT.

Regarding ECG the Echo and cardiac abnormality there were significant statistical differences in both levels of IL-1β and MDA.

Regarding pancreatic abnormality, there were insignificant statistical differences with both levels of IL-1β and MDA (Table 3).

**Table 3:** Showing comparison between level of IL-1β, MDA and patient demographic data, clinical examination including vital signs, GCS, biochemical investigations, cardiac and pancreatic functions

Parameter	IL-1β		MDA	
	*R*	*P* value	*R*	*P* value
Age (year)	-0.177	0.115	-0.181	0.106
Delay time (hour)	0.030	0.793	0.034	0.766
SBP (mmHg)	-0.175	0.119	-0.095	0.399
DBP (mmHg)	**-0.248**	0.026*	-0.155	0.166
Pulse (beat/min)	**0.427**	< 0.001*	**0.427**	< 0.001*
RR (cycle /min)	0.194	0.083	**0.261**	0.019*
GCS	**-0.439**	< 0.001*	**-0.514**	< 0.001*
Serum amylase (U/l)	0.125	0.266	**0.247**	0.026*
RBS (mg/ dl)	-0.023	0.837	-0.014	0.900
C TN- I (ng/ml)	**0.563**	< 0.001*	**0.594**	< 0.001*
Na+ (mmol/L)	**-0.454**	< 0.001*	**-0.570**	< 0.001*
K+ (mmol/L)	**-0.548**	< 0.001*	**-0.680**	< 0.001*
Urea (mg/dl)	-0.158	0.159	-0.089	0.431
Creatinine (mg/dl)	**0.411**	< 0.001*	**0.390**	< 0.001*
Hb (gm/dl)	-0.057	0.615	-0.051	0.648
TLC (cell/mm^3^)	-0.059	0.603	-0.192	0.086
PLT (cell/mm^3^)	0.152	0.174	**0.225**	0.044*
ECG abnormality	**0.591**	< 0.001*	**0.566**	< 0.001*
Echo abnormality	**0.555**	< 0.001*	**0.454**	< 0.001*
Cardiac abnormality	**0.591**	< 0.001*	**0.566**	< 0.001*
Pancreatic abnormality	0.182	0.104	0.182	0.104

-Pearson’s correlation*: Significant level at P value < 0.05, SBP: Systolic blood pressure, DBP: Diastolic blood pressure, RR: Respiratory rate, GCS: Glasgow Coma Scale, Na^+^: Sodium, K^+^: Potassium, Hb: Hemoglobin TLC: Total leukocytic count, PLT: Platelets ECG: Electrocardiogram, Echo: Echocardiography

### Linear regression analysis for factors affecting levels of IL-1β and MDA

Significant predictors for IL-1β were age, pulse, RR, GCS, NA^+^, K^+^, urea, creatinine, HB, PLT &echo. With highest standardized coefficient β were HB and pulse (Table 4).

Significant predictors for MDA were age, SBP, DBP, GCS, RBS, c TNI, K^+^, urea, HB, TLC, PLT, cardiac abnormality &echo. With highest standardized coefficient β were RBS, DBP & SBP (Table 4).

**Table 4:** Showing linear regression of patient demographic data, clinical examination including vital signs, GCS, biochemical investigations, cardiac and pancreatic functions and its effect on levels of IL-1β and MDA

Parameter	IL-1β		MDA	
	Standardized coefficient β	*P* value	Standardized coefficient β	*P* value
Age	0.17	0.016 *	0.26	< 0.001*
Delay time (hours)	0.15	0.11	0.042-	0.65
SBP (mmHg)	0.18-	0.23	0.51-	0.001*
DBP (mmHg)	0.14	0.38	0.54	0.001*
Pulse(beat/min)	0.81	< 0.001*	0.49	0.012*
RR (cycle/min)	0.33-	0.017*	0.064-	0.64
GCS	0.53	< 0.001*	0.27	0.038*
Serum amylase(U\I)	0.06-	0.52	0.12	0.17
RBS (mg \ dl)	0.04-	0.81	0.52-	0.001*
Troponin I(ng\ml)	0.21	0.027*	0.34	< 0.001*
Na+ (mmol \l)	0.18-	0.019*	0.09-	0.2
K+ (mmol\l)	0.56-	< 0.001*	0.46-	< 0.001*
Urea (mg\dl)	0.45-	< 0.001*	0.26-	0.009*
Creatinine (mg \dl)	0.33	0.003*	0.04-	0.71
Hb (g \dl)	1.02-	< 0.001*	0.48-	0.003*
TLC (cell\ cmm^3^)	0.28-	0.01	0.3-	0.007*
PLT (cell\ cmm^3^)	0.78-	< 0.001*	0.33-	0.019*
Echo abnormality	0.44	< 0.001*	0.27	0.005*
Cardiac abnormality	0.00	0.99	0.32	0.001*
Pancreatic abnormality	0.023	0.88	0.032	0.83

-linear regression*: Significant level at P value < 0.05, SBP: Systolic blood pressure, DBP: Diastolic blood pressure, RR: Respiratory rate, GCS: Glasgow Coma Scale, Na^+^: Sodium, K^+^: Potassium, Hb: Hemoglobin TLC: Total leukocytic count, PLT: Platelets, Echo: Echocardiography

### Cardiac function and IL-1β and MDA

There was significant difference between group A and B regarding their IL-1β and MDA levels (Table 5).

**Table 5:** Showing comparison between the cardiac function and IL-1β and MDA

Parameter	Cardiac function				*P* value
	Group A		Group B		
	*N* = 30		*N* = 51		
	Range	Mean ± SD	Range	Mean ± SD	
IL-1β (pg/ml)	(46.7–74.9)	57.2 ± 9.6	(49.1–89.3)	74.2 ± 12.3	< 0.001*
MDA (nmol/ml)	(1.5–2.8)	2 ± 0.5	(1.6–3.8)	2.8 ± 0.6	< 0.001*

-Independent Samples T test for parametric quantitative data between the two groups, *: Significant level at P value < 0.05, IL-1β: Interleukin-1β, MDA: Malondialdehyde

### Receiver operating characteristics (ROC) curve analysis of serum level of IL-1β and MDA for prediction of cardiac function abnormality

#### ROC curve analysis of serum level of IL-1β and MDA for prediction of cardiac function abnormality in at least one or more of cardiac function assessment parameters

**Fig. 1:**
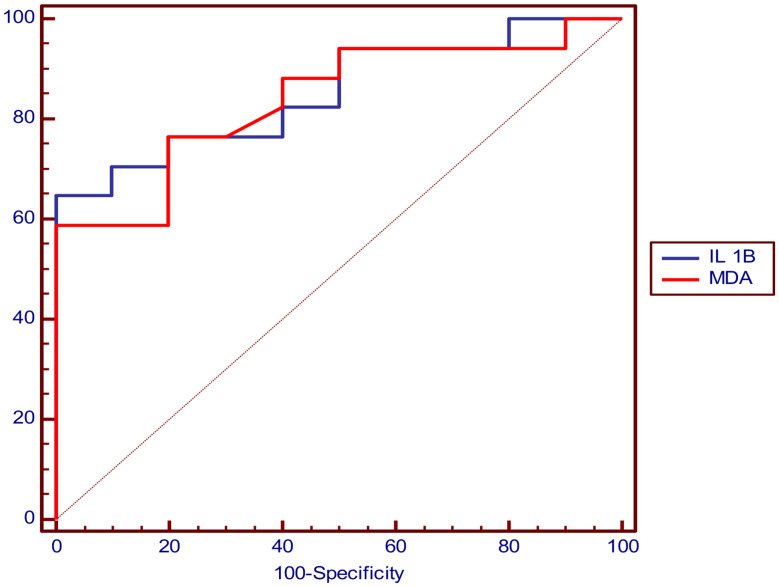
Showing ROC curve analysis of IL-1β and MDA in prediction of cardiac abnormality with AUC = 0.853 for IL-1β and = 0.838 for MDA

The cutoff value that can predict cardiac abnormality was > 50.5 pg/ml for IL-1β and > 1.77 nmol/ml for MDA and (Table 6; Fig. 1).

**Table 6:** Showing ROC curve analysis of IL-1β and MDA in prediction of cardiac function abnormality

Parameter	IL 1B	MDA
Optimal cutoff	> 50.5 (pg/ml)	> 1.77 (nmol/ml)
AUC	0.853	0.838
P value	< 0.001*	< 0.001*
Sensitivity	94.12%	94.12%
Specificity	50%	50%
PPV	76.2%	76.2%
NPV	83.3%	83.3%
Accuracy	77.8%	77.8%

-ROC: Curve analysis, AUC: Area Under curve, PPV: Positive Predictive Value, NPV: Negative Predictive Value, *: Significant level at P value < 0.05

#### Receiver operating characteristics (ROC) curve analysis of IL-1β and MDA in prediction of ECG abnormality

**Fig. 2:**
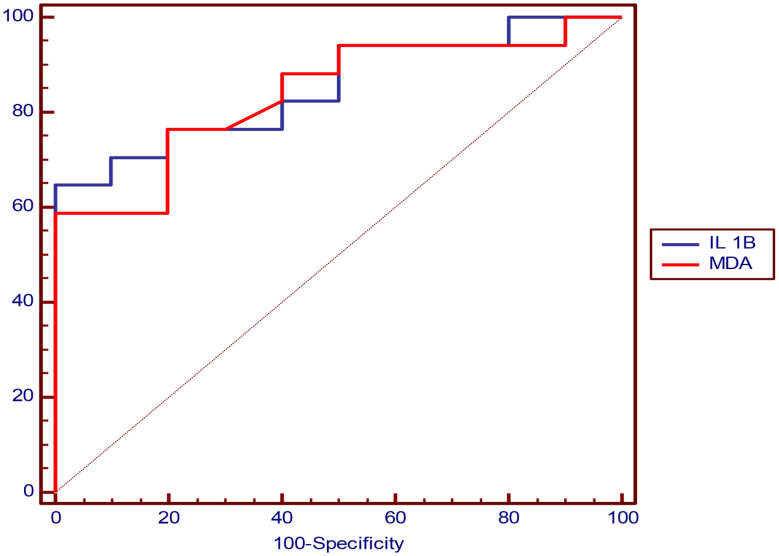
Showing ROC curve analysis of and IL-1β MDA in prediction of ECG abnormality with AUC = 0.853 for IL-1β and = 0.838 for MDA

The cutoff value that can predict ECG abnormality was > 50.5 pg/ml for IL-1β and > 1.77 nmol/ml for MDA and (Table 7; Fig. 2).

**Table 7:** Showing ROC curve analysis of IL-1β and MDA in prediction of ECG abnormality

Parameter	IL 1B	MDA
Optimal cutoff	> 50.5 (pg/ml)	> 1.77(nmol/ml)
AUC	0.853	0.838
P value	< 0.001*	< 0.001*
Sensitivity	94.12%	94.12%
Specificity	50%	50%
PPV	76.2%	76.2%
NPV	83.3%	83.3%
Accuracy	77.8%	77.8%

-ROC Curve analysis, AUC: Area Under curve, PPV: Positive Predictive Value, NPV: Negative Predictive Value, *: Significant level at P value < 0.05

#### ROC curve analysis of IL-1β and MDA in prediction of echo abnormality

**Fig. 3:**
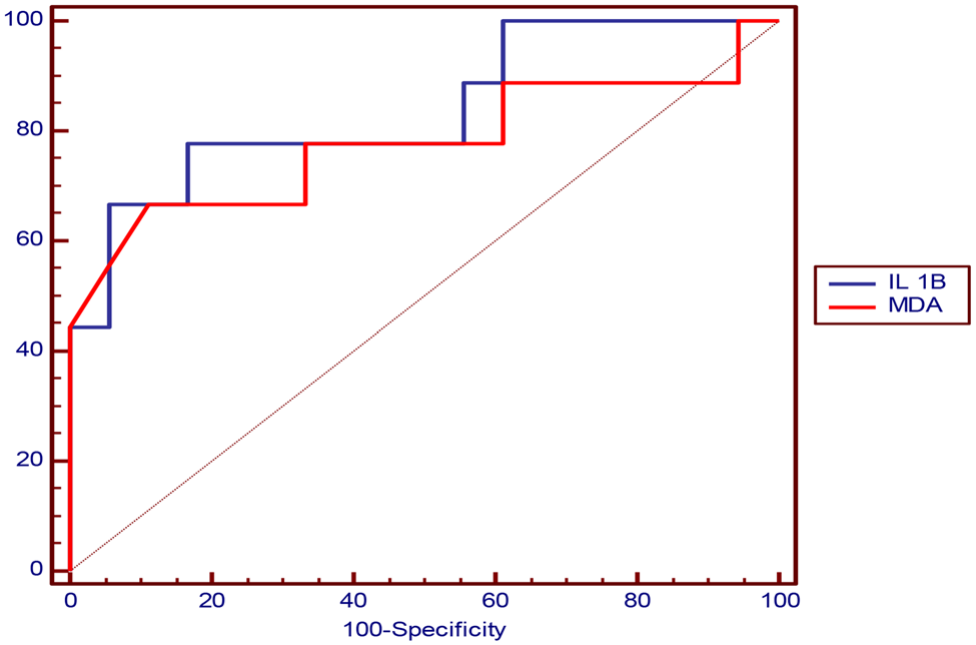
Showing ROC curve analysis of IL-1β and MDA in prediction of Echo abnormality with AUC = 0.840 for IL-1β and = 0.778 for MDA

The cutoff value that can predict Echo abnormality was > 78.11 pg/ml for IL-1β and = 2.95 nmol/ml for MDA and (Table 8; Fig. 3).

**Table 8:** Showing ROC curve analysis of IL-1β and MDA in prediction of echo abnormality

Parameter	IL 1B	MDA
Optimal cutoff	> 78.11 pg/ml	2.95 nmol/ml
AUC	0.840	0.778
P value	< 0.001*	< 0.001*
Sensitivity	66.67%	55.56%
Specificity	94.44%	94.44%
PPV	85.7%	83.3%
NPV	85%	81%
Accuracy	85.2%	81.5%

-ROC: Curve analysis, AUC: Area Under curve, PPV: Positive Predictive Value, NPV: Negative Predictive Value, *: Significant level at P value < 0.05

#### ROC curve analysis of IL-1β and MDA in prediction of c TN-I abnormality

**Fig. 4:**
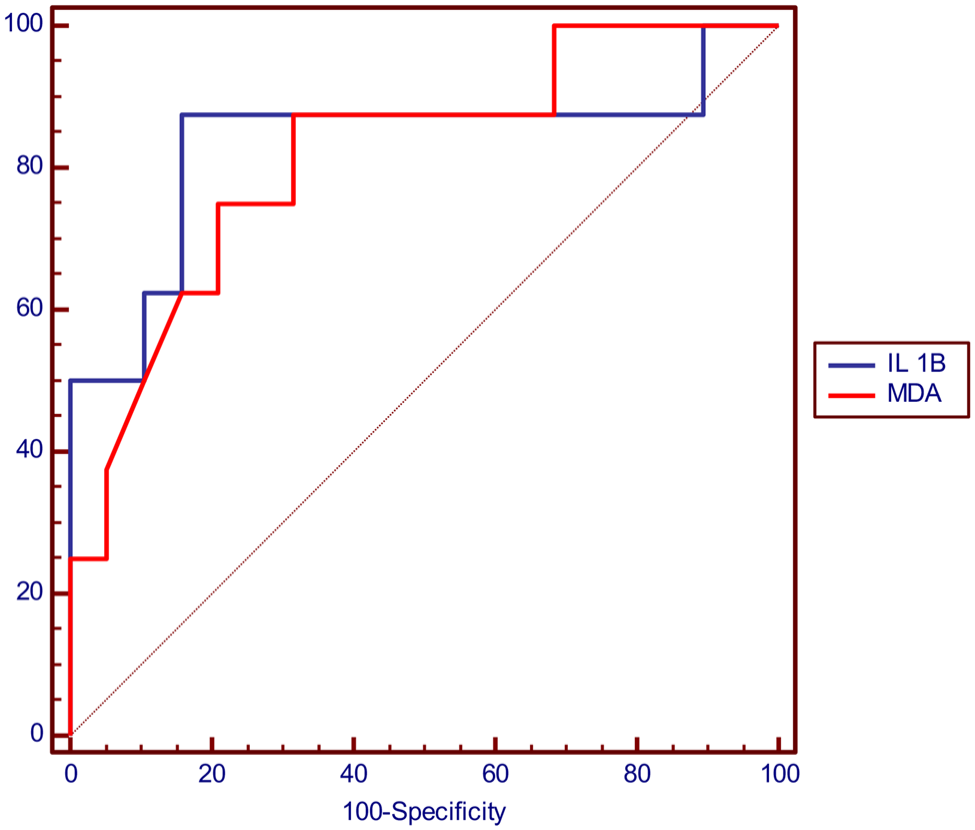
Showing ROC curve analysis of IL-1β and MDA in prediction of c TN-I abnormality with AUC = 0.836 for IL-1β and = 0.816 for MDA

The cutoff value that can predict c TN-I abnormality was > 81.5 pg/ml for IL-1β and > 2.81 nmol/ml for MDA and (Table 9; Fig. 4).

**Table 9:** Showing ROC curve analysis of IL-1β and MDA in prediction of c TN- I abnormality

Parameter	IL 1B	MDA
Optimal cutoff	> 81.5 pg/ml	> 2.81 nmol/ml
AUC	0.836	0.816
P value	< 0.001*	< 0.001*
Sensitivity	50%	75%
Specificity	100%	78.95%
PPV	100%	60%
NPV	82.6%	88.2%
Accuracy	85.2%	77.8%

-ROC: Curve analysis, AUC: Area Under curve, PPV: Positive Predictive Value, NPV: Negative Predictive Value, *: Significant level at P value < 0.05

### Pancreatic function and IL-1β and MDA

There was a significant statistical difference between pancreatic function with IL-1β and MDA (Table 10).

**Table 10:** Showing comparison between the patient pancreatic function with IL-1β and MDA

Parameter	Pancreatic function				*P* value
	Group C		Group D		
	*N* = 36		*N* = 45		
	Range	Mean ± SD	Range	Mean ± SD	
IL-1β (pg/ml)	(46.7–81.5)	64.3 ± 13.3	(48.8–89.3)	70.8 ± 14.1	0.038*
MDA (nmol/ml)	(1.5–3.1)	2.3 ± 0.6	(1.6–3.8)	2.6 ± 0.6	0.012*

-Independent Samples T test for parametric quantitative data between the two groups. Chi square test for qualitative data between the two groups, *: Significant level at P value < 0.05. N: number, IL-1β: Interleukin-1β, MDA: Malondialdehyde

### ROC curve analysis of serum level of IL-1β and MDA for prediction of pancreatic function abnormality

**Fig. 5:**
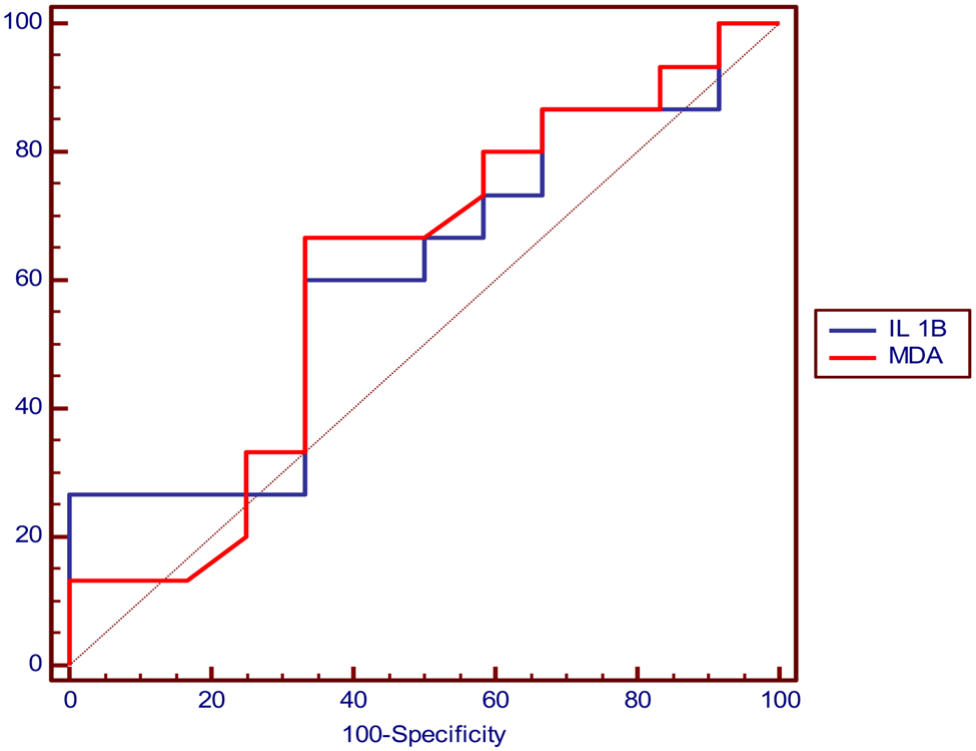
Showing ROC curve analysis of IL-1β and MDA in prediction of pancreatic abnormality with AUC = 0.606 for both

The cutoff value that can predict pancreatic function abnormality was > 50.5 pg/ml for IL-1β and > 2.54 nmol/ml for MDA and (Table 11; Fig. 5).

**Table 11:** Showing ROC curve analysis of IL-1β and MDA in prediction of pancreatic function abnormality

Parameter	IL 1B	MDA
Optimal cutoff	> 50.5 pg/ml	> 2.54 nmol/ml
AUC	0.606	0.606
P value	0.099	0.107
Sensitivity	86.67%	66.67%
Specificity	33.33%	66.67%
PPV	61.9%	71.4%
NPV	66.7%	61.5%
Accuracy	63%	66.67%

-ROC Curve analysis, AUC: Area Under curve, PPV: Positive Predictive Value, NPV: Negative Predictive Value, *: Significant level at P value < 0.05

## Discussion

Scorpions are arthropods of the Arachnids class. More than 1500 species of scorpions are found in the world, and their venom is a complex mixture of peptides, proteins, lipids, nucleotides, mucopolypeptides, biological amines, and other unidentified substances that may trigger toxicological and immunological reactions. Ion channel (Na, K, Ca, and Cl) blockers are the most important peptides in scorpion venom [14].

Following a scorpion sting, there may be mild, moderate, or severe signs and symptoms. Ranging from pain, nausea, and sweating up to heart failure, pulmonary edema, and even death in severe cases [15].

The sociodemographic variables in this study are in accordance with several studies which reported that acute scorpion poisoning was frequent in young adults [16] **.** Females were common in our study as the culture in upper Egypt suppose that females are responsible for cleaning and house works in their homes which located in rural parts near agricultural areas and some in Bedouin areas according to the nature of El-Minia city. Additionally, most stings occur inside of buildings and because it’s customary and traditional for women to stay inside as men wander outside [17].

Anemia was common among our patients, however normal TLC and PLT were recorded in more than two thirds of patients. A previous study revealed no significant hematological difference between poisoned subjects and normal values [18]. However, these findings disagreed with previous study that mentioned leukocytosis, thrombocytopenia and aberrant hematological values among the studied cases [19].

The variations can be attributed to the differences in scorpion venom compositions across diverse regions. Certain species release venom with cytotoxic and hemotoxic characteristics. Anemia may develop in these circumstances for multiple factors, including severe and life-threatening hemolysis, deep necrotic wounds, and coagulation issues [20].

Hyponatremia & hypokalemia in our patients is consistent with previous research [21]. Venom’s neurotoxic peptides target ion channels. Toxins alter the Na^+^ and K^+^ channel membrane conductance, which disrupts the neuronal influx and results in physio pathological diseases [22].

Normal renal functions in the studied cases are described in previous research [19, 23].

Regarding RBS, hyperglycemia with scorpion envenomation was reported in a systematic review in 2023 [24]. Hyperglycemia is accompanied by poor prognosis for scorpion stings as it can result in modifications to ECGs [19]. Pulmonary edema and cardiac dysfunction are closely correlated [25].

Dysregulation of hormones like glucagon and cortisol, as well as the suppression of insulin release, are influenced by the rise in catecholamines after scorpion envenomation. The development of clinical symptoms and multi-system organ failure is influenced by the rise in catecholamines, cortisol, and glucagon, which compete with the anabolic activities of insulin and prevent the organs from using glucose [26].

Serum amylases were normal in most patients. This was revealed in a prior retrospective study [27]. However, there are sporadic case studies about patients with high serum amylase level after scorpion envenomation [28].

There are two distinct processes through which the pancreas might be affected. The first occurs when toxin types bind to voltage-gated sodium channels, fixing the channels in an open state and inhibiting potassium channels. The second process inactivates the exocrine pancreas by releasing calcium from intra-acinar reserves [28, 29].

A recent study on mice revealed the correlation between scorpion envenomation and hyperglycemia. The authors supposed that venom enhances the release of IL-1α and IL-1β in the pancreas, which will subsequently release nitric oxide (NO) and induce β islets cells edema. This pathway is suggested to increase glucose release during severe scorpion poisoning. In the mouse model of poisoning, survival and clinical symptoms were improved by blocking or not having the IL-1R receptor they supported addition of NO production inhibitors in addition to antiserum to treat hyperglycemia [12].

Since the pancreas is exposed to high levels of scorpion venom in the first 24 h following the incident and scorpion venom causes an exacerbated inflammatory response in various organs, it was hypothesized that the immune response also contributed to pancreatic dysfunction [30].

The insignificant correlation in our study with pancreatic dysfunction detected may be attributed to the existence of different pathways rather than the inflammatory pathway which may predominate in some patients like autonomic storm. Rapid escalation in free fatty acids (FFA) levels because of catecholamine related lipolysis contributes to insulin resistance [31]. The massive release of neurohormones can also result in hyperamylasemia which is also strongly correlated to interleukin 6 (IL6) [32]. It is also predictable that the scorpion venom components can vary around the world which may give rise to diverse outcomes [33].

More than half of our cases had elevated c TN I. Previous studies have mentioned elevated serum c TN I with scorpion envenomation [34], and some revealed that the peaks were not initial and achieved 24–36 h after being bitten by a scorpion [35].

Systemic inflammatory response syndrome, which has been linked to a notable elevation in troponin, can be linked to scorpion envenomation, even if the time between envenomation and arrival to the emergency department is short [36].

ECG changes were detected and declared to be regular among scorpion sting victims [24]. Abnormal echo studies are recurrent findings like mitral regurge (MR), tricuspid regurge (TR) and abnormal motion [37].

Scorpion-related cardiomyopathy can be described as a reversible status characterized by a significant disruption in the function of the right and left ventricles. The vascular phase starts, which is characterized by systemic elevated blood pressure, with subsequent acutely elevated LV afterload, and vasoconstriction brought on by catecholamine release in circulation. LV-filling pressure upsurges due to hypertension, which hinders LV emptying. Reduced LV contractility follows, which results in a low cardiac output and shock stage. Right ventricular dysfunction occurs symmetrically to LV dysfunction in this stage, which is likewise reversible. With inotropic therapy or spontaneous reversal, this phase may return to normal. As a result of epicardial coronary vasospasms and jumping in coronary artery resistance, scorpion related cardiomyopathy may exhibit symptoms of a rise in catecholamines which would present as myocardial ischemia [1, 38].

The ECG changes may occur due to cardiogenic pulmonary edema associated with catecholaminergic activity which declines myocardial perfusion due to micro-vascularture spasm, and the direct effect of venom on myocardial fibrils [39].

An obvious rise in levels of IL-1β were detected in sera of different mouse strains injected with scorpion venom [40, 41].

The only human study found to document the direct evidence that serum IL-1β is significantly elevated after scorpion sting, correlates with severity, and declines in survivors after 24 h. They declared that non-survivors maintained high IL-1β levels, suggesting a link between persistent inflammation and poor outcomes [36].

In comparison to our study, they did not evaluate the possibility of incorporation of these data in clinical management of scorpion envenomation and when to administer anti-inflammatory as adjunct therapy.

Precise risk stratification and management decisions in emergency department regarding scorpion envenomed patients’ disposition and the appropriate level of care assignment depend critically on identification of cardiac involvement. The common cardiac clinical assessment methods are often not accurate in detection of subtle cardiac dysfunction, particularly in borderline presentations in case of tachypnea and/or excessive bronchial secretions. We can emerge evidence suggests that adjuvant therapies to antivenom administration may significantly improve outcomes in severe cases. In this context, biochemical markers of myocardial injury serve as valuable diagnostic tools, enabling earlier detection of cardiac compromise and facilitating more informed clinical decision-making regarding patient management [42, 43].

Unlike other cytokines as IL-6, IL-8, IL-10 and TNF-α which are involved in scorpion envenomation, the IL-1β is different as it is directly activated by the venom products. Scorpion venom (e.g., from Androctonus, Tityus, Leiurus species) composed of neurotoxins (e.g., Na⁺/K⁺ channel modulators) that directly enhance activity of the NOD-like receptor family pyrin domain-containing 3 (NLRP3) inflammasome, and the result is caspase-1-dependent IL-1β release [41].

Additionally, Scorpion venom is recognized by macrophages which activate inflammatory response and release IL-1β, which enhance lung edema and death, as Interleukin-1 receptor induces prostaglandin E2 (PGE2) production which mediates acetylcholine correlated cardiac function irregularities and induction of mortality. As it is proved cytokine can desensitize cardiomyocytes following adrenergic stimulation and cause reversible contractile dysfunction in the musculature of the heart in association with arrhythmias. A study considered the need to consider the contribution of PGE2 and IL-1β, which are released systemically alongside by the lungs, to cardiac function abnormality in addition to the mediators released by the heart cells is mandatory. The authors strongly advocated for early dexamethasone administration, even preceding antiserum treatment, to inhibit inflammatory mediator and acetylcholine release. By that they could reduce mortality associated with scorpion envenomation induced cardiac dysfunction [11].

It was noticed that direct human studies on MDA and the impact of antivenom on these biomarkers are lacking. Most evidence is from animal models, with human data limited to clinical observations and laboratory abnormalities. That creates a research gap between proved MDA incorporation in scorpion envenomation in animal models and existence of a documented correlation in human studies. In this setting we were trying to investigate the role of administration of antioxidants as adjunct therapy in improving outcomes for cardiac and pancreatic abnormalities.

There were significant statistical differences with levels of MDA regarding cardiac abnormality. There were insignificant statistical differences with MDA regarding RBS and serum amylase. The highest significant predictors for MDA by linear regression were RBS, DBP & SBP.

Lipid peroxidation was assessed in scorpion envenomation in previous studies on rats. A significant elevation in MDA level in the lung, heart, liver, and kidneys of envenomed animals was described [7, 44].

A previous study revealed a significant elevation in MDA level among scorpion envenomed subjects than the controls with no correlation between cardiac dysfunction and lipid peroxidation markers MDA. They explained that the absence of correlation may be due to the relatively small sized sample. As the level of lipid peroxidation marker was significantly higher in envenomed than the non-envenomed which correlates with the induction of myocardial injury by scorpion venom and is accompanied by a shift to oxidative status [45].

Regarding peroxidation in pancreatic and liver tissues, a significant elevation of MDA levels in envenomed rats was detected in previous research concerning pancreatic and liver tissue homogenates. It was hypothesized that the improvement of antioxidant status with decrease of MDA and nitrite levels in the treated animals’ groups resulted in improving rats’ fate in the study [46].

lipid peroxidation in tissues escalates NO and reactive oxygen species levels. Fatty acids in cell membranes can indeed react with nitrogen and oxygen species to compromise their function. The MDA, which is the main byproduct of lipid peroxidation, may intensify the immunopathology response [47].

## Conclusion

It could be concluded that there were significant statistical differences with both levels of IL-1β and MDA regarding cardiac abnormality. There were insignificant statistical differences with both levels of IL-1β and MDA regarding RBS and serum amylase. IL-1β level > 50.5 pg/ml is associated with a probability of developing cardiac dysfunction in scorpion poisoning victims. The patient with cutoff value of IL-1β > 78.11 pg/ml have a probability of developing echo abnormality. The patient with a cut off value of IL-1β > 81.5 pg/ml have a probability of developing c TN-I abnormality.

IL-1β level > 50.5 pg/ml is associated with a probability of developing pancreatic dysfunction in one or both of RBS and serum amylase.

The highest significant predictors for IL-1β by linear regression were HB and pulse. And for MDA were RBS, DBP & SBP.

Establishing specific cutoff values enables toxicologists to evaluate the pathophysiological mechanisms underlying cardiac and pancreatic dysfunction following scorpion envenomation. These biomarkers can guide accurate administration of NO inhibitors and anti- inflammatory agents as dexamethasone alongside antivenom therapy, which is considered to reduce the potential complications and mortality rates.

### Limitations

More studies that correlate pancreatic dysfunction in scorpion poisoned patients and the inflammatory and oxidative stress pathways are recommended to be incorporated in clinical practice.

More animal and human studies needed to evaluate efficacy of NO inhibitors and anti- inflammatory agents alongside antivenom therapy.

Higher sample size studies are expected to be more beneficial.

Studies on the role of IL-1β and MDA in cardiac and pancreatic abnormalities development specified to different scorpion species around universe may be more beneficial and result in various outcomes.

## Data Availability

The datasets generated and/or analyzed during the current study are not publicly available due to patients’ privacy (investigations reports contain names and private data) but are available from the corresponding author on reasonable request.

## Abbreviations

AUC: Area Under curve
Bpm: Beat per minute
CBC: Complete blood count
Mm^3^: Cubic Millimeter
C TN-I: Cardiac troponin I
GCS: Galsgow Coma Scale
DBP: Diastolic blood pressure
ECG: Electrocardiography
ECHO: echocardiography
EDTA: Ethylenediaminetetraacetic acid
ELISA: Enzyme linked immunosorbent assay
g/dl: Gram per decilitre
HB: Hemoglobin
IL1: Interleukin 1
IL-1R: Interleukin 1recepror
IL-1β: Human Interleukin 1 Beta
IL-6: Interleukin-6
K^+^: Potassium
LV: Left ventricle
MDA: Malondialdehyde
mg/dl: Milligrams per decilitre
mmHg: Millimeters of mercury
mmol/L: millimoles per liter
MR: Mitral regurge
N: Number
Na^+^: Sodium
ng/ml: Nanogram per milliliter
nm: Nano mole
nmol/ml: Nanomole per milliliter
NO: Nitric oxide
NPV: Negative Predictive Value
OD: Optical density
pg/ml: Picogram per milliliter
PGE2: Prostaglandin E2
PLT: Platelets
PPV: Positive Predictive Value
RBS: Random blood sugar
ROC Curve: Receiver Operating Characteristic
ROS: Reactive oxygen species
RR: Respiratory rate
RV: Right ventricle
SBP: Systolic blood pressure
SD: Standered deviation
SPSS: Statistical Package for Social Sciences
TLC: Total leucocytic count
TR: Tricuspid regurge
U/l: Unit per liter
